# Differential Anatomical Expression of Ganglioside GM1 Species Containing d18:1 or d20:1 Sphingosine Detected by MALDI Imaging Mass Spectrometry in Mature Rat Brain

**DOI:** 10.3389/fnana.2015.00155

**Published:** 2015-12-01

**Authors:** Nina Weishaupt, Sarah Caughlin, Ken K.-C. Yeung, Shawn N. Whitehead

**Affiliations:** ^1^Department of Anatomy and Cell Biology, Schulich School of Medicine and Dentistry, University of Western Ontario, LondonON, Canada; ^2^Department of Chemistry and Department of Biochemistry, Schulich School of Medicine and Dentistry, University of Western Ontario, LondonON, Canada

**Keywords:** MALDI, imaging mass spectrometry, GM1, ganglioside expression, cortex

## Abstract

GM1 ganglioside plays a role in essential neuronal processes, including differentiation, survival, and signaling. Yet, little is known about GM1 species with different sphingosine bases, such as the most abundant species containing 18 carbon atoms in the sphingosine chain (GM1d18:1), and the less abundant containing 20 carbon atoms (GM1d20:1). While absent in the early fetal brain, GM1d20:1 continues to increase throughout pre- and postnatal development and into old age, raising questions about the functional relevance of the GM1d18:1 to GM1d20:1 ratio. Matrix-assisted laser desorption/ionization imaging mass spectrometry is a novel technology that allows differentiation between these two GM1 species and quantification of their expression within an anatomical context. Using this technology, we find GM1d18:1/d20:1 expression ratios are highly specific to defined anatomical brain regions in adult rats. Thus, the ratio was significantly different among different thalamic nuclei and between the corpus callosum and internal capsule. Differential GM1d18:1/GM1d20:1 ratios measured in hippocampal subregions in rat brain complement previous studies conducted in mice. Across layers of the sensory cortex, opposing expression gradients were found for GM1d18:1 and GM1d20:1. Superficial layers demonstrated lower GM1d18:1 and higher GM1d20:1 signal than other layers, while in deep layers GM1d18:1 expression was relatively high and GM1d20:1 expression low. By far the highest GM1d18:1/d20:1 ratio was found in the amygdala. Differential expression of GM1 with d18:1- or d20:1-sphingosine bases in the adult rat brain suggests tight regulation of expression and points toward a distinct functional relevance for each of these GM1 species in neuronal processes.

## Introduction

**FIGURE 1:**
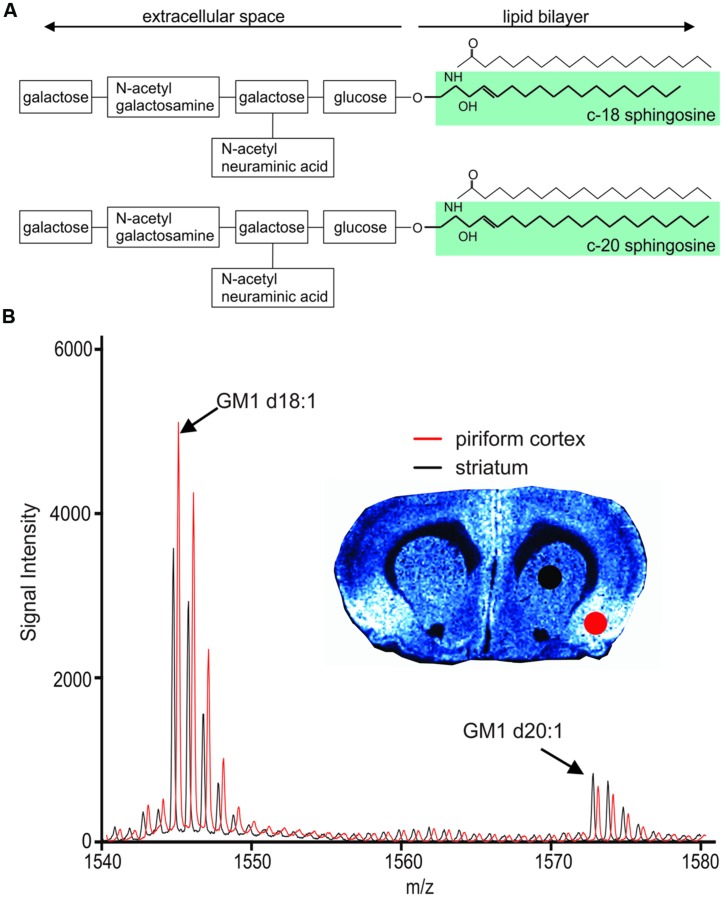
**Chemical structure of GM1 d18:1 (**A**, top) and GM1 d20:1 (**A**, bottom).** Mass spectra in the GM1 m/z range generated from two different anatomical regions, the piriform cortex and the striatum, show differential expression of GM1d18:1 and GM1d20:1. **(B)** The spectrum shown in red has been shifted to the right for better visualization of differences in peaks. Molecular image shows expression of GM1d18:1. For all analyses, the area under the curve (AUC) was calculated for the highest peak of each species (arrows).

Gangliosides are sialylated glycosphingolipids composed of a hydrophobic ceramide base anchored within the cellular membrane and a carbohydrate moiety that extends into the extracellular space (**Figure 1A**). Gangliosides are highly clustered within lipid rafts, specialized membrane microdomains where signaling molecules are abundant and where the lipid composition can greatly influence the accessibility of proteins involved in signal transduction and cell metabolism (Sonnino et al., 1990; Sonnino and Prinetti, 2010). It is therefore not surprising that gangliosides are especially prominent in brain tissue, accounting for about ten percent of the brain’s lipid mass. Among gangliosides, GM1 is highly expressed within the brain and is known to be involved in a number of neuronal functions, including neuronal differentiation, survival, neurotransmission and neuritogenesis (Posse de Chaves and Sipione, 2010; Ledeen and Wu, 2015). Interest in GM1 has peaked in recent years for its therapeutic potential in neurodegenerative conditions, namely Huntington’s disease (Maglione et al., 2010; Di Pardo et al., 2012) and Parkinson’s disease (Schneider et al., 2010). Yet, there is also a substantial body of evidence linking GM1 to the production of amyloid beta fibrils in Alzheimer’s disease (Ueno et al., 2014; Yanagisawa, 2015).

While most research on gangliosides in neuroscience has focused on their carbohydrate moiety, little is known about the relevance of different sphingosine chain lengths, which are not readily accessible by immunolabeling techniques. Using chromatographic and mass spectrometric approaches, several distinct species of GM1 have been identified based on the long chain base of their ceramide moiety. GM1 containing 18 carbon atoms in its sphingosine base, hereafter referred to as GM1d18:1, is the predominant species in the brain and detectable already in early fetal development (Rosenberg and Stern, 1966). GM1 with 20 carbon atoms in its sphingosine base, hereafter referred to as GM1d20:1, is not detectable in early development but is increasingly expressed during pre- and postnatal development (Rosenberg and Stern, 1966; Sonnino and Chigorno, 2000). While GM1d18:1 remains the dominant species, aged brains show an increase in GM1d20:1 compared to young brains (Sonnino and Chigorno, 2000). The developmental difference in the expression of GM1d18:1 and GM1d20:1 has recently been confirmed in the mouse hippocampus with an IMS approach similar to the one used in this study, where a differential expression of the two species was also evident in anatomical subregions of the hippocampus (Sugiura et al., 2008). These findings indicate that the expression of GM1 containing different sphingosine bases is regulated tightly both temporally and spatially.

While the temporal evolution of GM1d18:1/GM1d20:1 ratio throughout development and maturation of the brain is well described (Sonnino and Chigorno, 2000; Sugiura et al., 2008), little is known about the spatial distribution of GM1d18:1 and GM1d20:1 in different brain regions, outside the hippocampus. A novel and emerging technique, MALDI IMS can identify GM1 species with different ceramide moieties, and allows quantification of their expression within anatomical context (Goto-Inoue et al., 2011; Whitehead et al., 2011; Ellis et al., 2013). Using this technique, we find GM1d18:1/GM1d20:1 ratios are specific to anatomically distinct brain regions, suggesting a functional relevance of the so far largely neglected ceramide moiety of gangliosides in the brain. Our findings emphasize that new technologies may uncover novel meaningful biological processes that were previously unknown due to the bias in knowledge toward what can be reliably detected with the tools of the time.

## Materials and Methods

### Procedures Involving Live Animals

All procedures involving live animals were in accordance with the guidelines of the Canadian Council on Animal Care and approved by the University of Western Ontario Animal Use Committee (Protocol 2014–2016). Twenty-three female Fisher rats, which were part of an in-house breeding colony, were used at 8 to 10 months of age at euthanasia. These rats were part of an experiment to answer the question whether stroke changes ganglioside expression differentially in WT rats compared to rats that overexpress APP (an early Alzheimer’s Disease model). As no statistically significant changes in ganglioside expression were detected in any experimental group (**Supplementary Figure S1**), all animals were pooled for the anatomical analyses presented in this article. All rats were bred in house. Founder breeding pairs were kindly provided by Dr. Yuksel Agca (University of Missouri). Fourteen animals were homozygous TG for the human APP gene carrying both the Swedish and Indiana mutations (Agca et al., 2008). Eight WT and eight TG animals received a small experimental stroke by stereotaxic injection of 3 μl saline containing 10 pmol (ET-1, Sigma–Aldrich, St. Louis, MO, USA) into the right striatum (+0.5 mm anterior, 3 mm lateral to bregma at 5 mm depth below the dura) 28 days before euthanasia (Caughlin et al., 2015). The remaining one WT and six TG animals did not undergo surgical procedures. All animals were euthanized with an overdose of pentobarbital (Euthanyl), brains were harvested without delay, the cerebellum removed, and the rest of the brain was frozen on dry ice and stored at -80°C.

### Tissue Sample Preparation

Brains were attached to a specimen holder on dry ice with distilled water. Using a cryostat, 10 μm thick coronal brain sections were thaw-mounted onto indium tin oxide (ITO) coated glass slides (Hudson Surface Technology Inc., Old Tappan, NY, USA). Slides were dehydrated for 15 min in a vacuum chamber and then coated with 1,5-diaminonaphthalene (DAN, Sigma–Aldrich, Oakville, ON, Canada) matrix using a sublimation apparatus (Chemglass Life Sciences, Vineland, NJ, USA). DAN matrix was sublimated for 9 min at 130°C sand bath temperature. Following matrix application, a calibration mixture of five standard proteins (4700 Calibration Mixture, AB Sciex, Concord, ON, Canada) was mixed with alpha-cyano-4-hydroxy-cinnamic acid matrix (CHCA, Sigma–Aldrich) and 0.75 μl spots were applied on DAN matrix-free areas of the glass slide in the vicinity of the section to be imaged. Once calibration spots had dried, slides were stored at -20°C overnight. The following day, glass slides were thawed and dehydrated in a vacuum chamber for 15 min.

### MALDI Imaging Mass Spectrometry

Indium tin oxide coated glass slides with sample sections were mounted into a MALDI holding plate and inserted into a Sciex MALDI 5800 TOF/TOF mass spectrometer (Sciex, Framingham, MA, USA). Following calibration at 50 ppm mass tolerance, image acquisition was started in reflectron and negative ion mode, with a 70 μm laser step distance. A mass spectrum with a 1000–2000 m/z range was acquired for each laser shot.

### Analysis of Molecular Image Data

**FIGURE 2:**
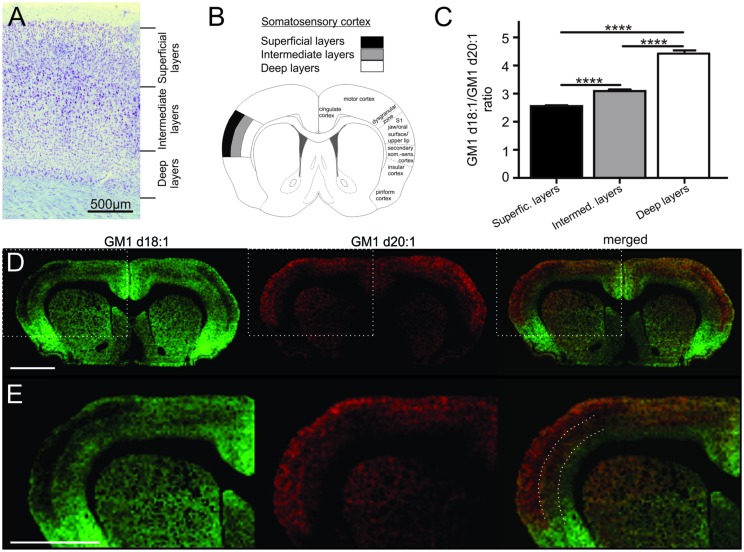
**Cortical sampling areas are depicted in a histological section stained with thionine **(A)**.** Mass spectra were generated from a superficial, an intermediate and a deep region of interest within the somatosensory cortex (**B**, modified from Paxinos and Watson, 1998), and corresponding regions were also sampled in two more posterior sections per animal. GM1d18:1/GM1d20:1 ratios were significantly different (Tukey’s multiple comparisons, ^∗∗∗∗^*p* < 0.0001) among cortical layers **(C)**. Molecular images show the anatomical expression of GM1d18:1 (green) and GM1d20:1 (red) in the cortex **(D)**. Scale bar = 3 mm. Dashed box marks cortical region magnified in bottom row **(E)**. Dashed lines in composite image mark the borders between sampling areas for different cortical layers. Scale bar = 3 mm. All images are optimized for visualization and do not represent absolute concentrations (GM1d18:1 is the predominant species even in the superficial layers of the cortex).

Molecular images were analyzed using Tissue View Software (Sciex). All images were optimized for visualization of expression, therefore color intensities do not reflect the same absolute signal intensity across different images. In each section, similar regions of interests (ROIs) were drawn for each anatomical region in the left and right hemisphere. As we cannot identify anatomically defined regions based on their cytoarchitecture in molecular images, we selected ROIs with reference to the Rat Brain Atlas by Paxinos and Watson (1998). The sampled cortical regions include putative primary and secondary somatosensory cortex (**Figure 2B**). The average mass spectral data for each ROI was exported, and the baseline noise was removed for each peak. GraphPad Prism software version 6 (GraphPad Software Inc., La Jolla, CA, USA) was used to measure the area under the curve (AUC) to quantify the highest peak for GM1d18:1 at a predicted m/z of 1544.87, and for GM1d20:1 at a predicted m/z of 1572.9, respectively (**Figure 1B**). Subsequently, the ratio of AUC of the highest GM1d18:1 peak to that of the highest GM1d20:1 peak was calculated for each ROI. Based on previous studies (Whitehead et al., 2011; Caughlin et al., 2015) measuring the AUC for the first (largest) peak is indicative of total signal for all isotope peaks of the species. Four measurements were taken for each animal (both hemispheres in two sections), and the average per animal was presented in graphs and used for statistical analyses. The corpus callosum and cortical layers were sampled in eight ROIs (in two anterior and two posterior sections, in both hemispheres). In cases where image resolution was insufficient for detailed analysis, the average value was taken from less than four sample measurements. Individual animal’s ROIs with less than two measurements were excluded. Each ROI is represented by average values from 16 to 20 brains. All calculated GM1d18:1/GM1d20:1 ratios are available in the MetaboLights database (MTBLS271).

### Statistical Analyses

Statistical comparisons were performed using GraphPad Prism software (GraphPad Software Inc.). Data sets were first tested for fitting a Gaussian distribution, and based on the result, either a *t*-test or a Mann–Whitney test was used to compare two anatomical regions, and a one-way ANOVA with Tukey’s multiple comparisons or a Kruskal–Wallis test was performed to compare the GM1d18:1/GM1d20:1 expression ratio among three or more anatomical regions. Bar graphs depict the mean and the standard error of the mean (SEM), which is also stated in the text. A *p* value of <0.05 was considered significant.

## Results

### GM1d18:1/GM1d20:1 Ratio in Different Experimental Conditions

In order to detect any potential differences in the GM1d18:1/GM1d20:1 ratio between WT and TG animals, as well as between animals that received a low-dose striatal ET-1 injection and controls, the GM1d18:1/GM1d20:1 ratio was plotted for all experimental groups for each region. There was no significant difference among the groups in any one region analyzed here (**Supplementary Figure S1**), which allowed us to pool all experimental animals to increase the power for the anatomical study. The low variability among individual GM1d18:1/GM1d20:1 ratios in each region was reflected by the small SEMs.

### GM1d18:1/d20:1 Ratio within the Cerebral Cortex

The cerebral cortex is divided into six layers based on cellular morphology and function (**Figure 2A**). While individual layers were not clearly distinguishable in molecular images, a gradient of expression of both GM1 species analyzed across the depth of the somatosensory cortex was observed (**Figure 2B**). Superficial layers had relatively low GM1d18:1 expression and relatively high GM1d20:1 signal intensities, intermediate layers had an intermediate signal intensity for both GM1 species, and the deep layers showed substantial GM1d18:1 expression, while lacking considerable amounts of GM1d20:1 (**Figures 2C–E**). When quantified, these opposing expression gradients for both GM1 species (**Figure 2E**) resulted in a significantly lower GM1d18:1/GM1d20:1 ratio in the superficial layers (2.55 ± 0.03, *n* = 14) compared to deeper layers (Tukey’s multiple comparisons, *p* < 0.0001, **Figure 2C**). In contrast, the ratio in the deepest layers was significantly higher (4.43 ± 0.11, *n* = 14) than in all other layers (Tukey’s multiple comparisons, *p* < 0.0001) and almost twice as high as the ratio measured in superficial layers (**Figure 2C**). These measurements were consistent between layers of the somatosensory cortices in sections anterior to bregma and layers of the somatosensory cortices at about 3 mm posterior to bregma. The average of all analyzed regions is shown (**Figure 2C**). When comparing cortical areas neighboring the sampled cortical regions to the GM1d18:1/GM1d20:1 expression pattern quantified above, it was obvious that this is not a pattern consistent throughout all cortical areas and layers. There were clearly visible borders to this expression pattern, with the superficial layers of the dorsal motor cortex and the cingulate cortex expressing relatively more GM1d18:1 than the somatosensory cortex expresses in its superficial layers (not quantified). The molecular images also showed a strong GM1d18:1 dominance in the piriform cortex neighboring the somatosensory cortex ventrally. The piriform cortex, which is largely devoid of GM1d20:1, is considered archicortex and is evolutionarily among the oldest cortical areas.

### GM1d18:1/d20:1 Ratio within Major White Matter Tracts

**FIGURE 3:**
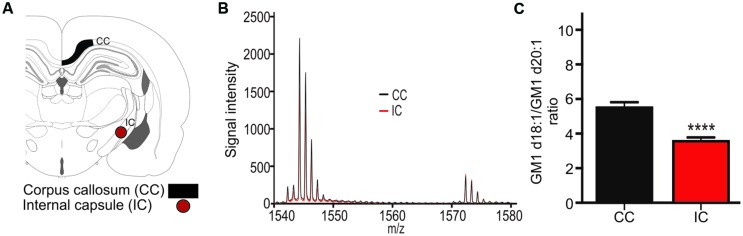
**A schematic cross section shows the ROIs used to generate mass spectra for the corpus callosum (CC), which was also sampled in more anterior sections, and the internal capsule (IC, **A**).** Mass spectrum from a representative section shows differential expression of GM1d18:1 And GM1d20:1 in the CC and IC **(B)**. The GM1d18:1/GM1d20:1 ratio was significantly lower in the IC than in the CC (Mann–Whitney test, ^∗∗∗∗^*p* < 0.0001, **C**).

The expression ratio of the two GM1 species was measured in the corpus callosum (CC, **Figure 3A**) and in the internal capsule (IC, **Figure 3A**). GM1 signal was so low in the white matter relative to surrounding gray matter that meaningful images could not be generated, however, mass spectra clearly indicated the presence of both GM1d18:1 and GM1d20:1 within the sampled white matter regions (**Figure 3B**). The average ratio of all sampling areas per animal for the CC (5.506 ± 0.31, *n* = 19) was significantly higher than the ratio within the internal capsule (3.56 ± 0.22, *n* = 18, Mann–Whitney, *p* < 0.0001, **Figure 3C**).

### GM1d18:1/d20:1 Ratio within the Hippocampus

**FIGURE 4:**
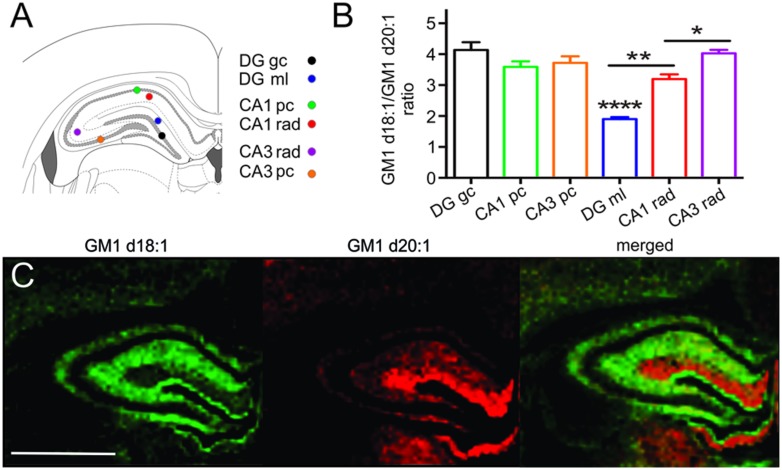
**Schematic shows the small sampling points within the hippocampus from which mass spectra were generated **(A)**.** While the cell layers across the CA1, CA3, and DG had a similar GM1d18:1/GM1d20:1 ratio, the ratio differed significantly among the molecular layer of the DG and the stratum radiatum in the CA1 and CA3 regions (Kruskal–Wallis test, DG vs. CA1 ^∗∗^*p* < 0.001, DG vs. CA3 ^∗∗∗∗^*p* < 0.0001, CA1 vs. CA3 ^∗^*p* < 0.05, **B**). CA1 pc, CA1 PC; CA1 rad, CA1 stratum radiatum; DG gc, dentate gyrus GC; DG ml, dentate gyrus molecular layer; CA3 rad, CA3 stratum radiatum; CA3 pc, CA3 PC. Molecular images of GM1d18:1 expression (green) and GM1d20:1 expression (red) showed high expression of the d20:1-species almost exclusively within the DG ml, while the d18:1-species was highest within the stratum radiatum **(C)**. Scale bar = 2 mm.

Sampling regions for spectral analysis were considerably smaller in hippocampal areas than in all other brain regions to limit accidental inclusion of neighboring regions, especially when sampling the narrow cellular layers (**Figure 4A**). Among the cell layers of the DG, (4.14 ± 0.25, *n* = 20), CA field 1 (CA1, 3.59 ± 0.18, *n* = 20) and CA field 3 (CA3, 3.72 ± 0.21, *n* = 17), there was no statistical difference in the GM1d18:1/GM1d20:1 expression (**Figure 4B**). However, the GC of the DG has a slightly higher ratio than the PCs of CA1 and CA3 (**Figure 4B**). In contrast, we found an extremely low GM1d18:1/GM1d20:1 ratio within the molecular layer of the DG (1.90 ± 0.06, *n* = 21, **Figure 4C**). This layer stood out as the area of greatest relative GM1d20:1 expression in a cross section of the hippocampus, while GM1d18:1 expression was visibly lower in this layer than in the neighboring stratum radiatum (**Figure 4C**). The GM1d18:1/GM1d20:1 ratio within the molecular layer of the DG is significantly lower than the ratio within the stratum radiatum of the CA1 (3.19 ± 0.15, *n* = 20) and CA3 region (4.03 ± 0.12, *n* = 21, Kruskal–Wallis test, DG vs. CA1 *p* < 0.001, DG vs. CA3 *p* < 0.0001, **Figure 4B**). Careful visual examination of the GM1d20:1 expression image (red, **Figure 4C**) indicated a change in expression between the stratum radiatum in the CA1 versus the CA3 region, which was reflected by a significantly lower GM1d18:1/GM1d20:1 ratio in the CA1 region (Kruskal–Wallis test, *p* < 0.05, **Figure 4B**).

### GM1d18:1/d20:1 Ratio in the Thalamus

**FIGURE 5:**
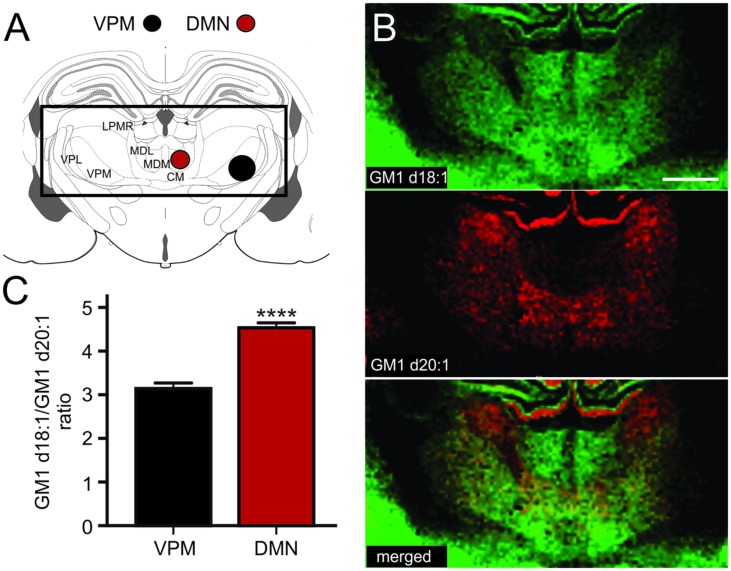
**Sampling regions for the DMN included its lateral and medial parts, the ROI covering the VPM may also have included parts of the VPL **(A)**.** CM, central medial thalamic nucleus; LPMR, lateral posterior thalamic nucleus, mediorostral part; MDL, mediodorsal thalamic nucleus, lateral part; MDM, mediodorsal thalamic nucleus, medial part; VPL, ventral posterolateral thalamic nucleus; VPM, ventral posteromedial thalamic nucleus. Molecular images of GM1d18:1 expression (green) and GM1d20:1 expression (red) showed differential expression of the two species within the DMN and VPM, as well as within the LPMR (not quantified, **B**). Scale bar = 1 mm. The ratio of GM1d18:1/GM1d20:1 was significantly higher within the DMN than the VPM (Mann–Whitney test, ^∗∗∗∗^*p* < 0.0001, **C**).

The thalamus is a highly diverse structure that offers a multitude of regions for analysis of GM1 expression. Because we can sample areas that are visibly delineated by their GM1d18:1 or GM1d20:2 expression profile with the highest confidence, we chose the DMN and the ventral posteromedial nucleus (VPM) of the thalamus for analysis (**Figure 5A**). While the DMN demonstrated high GM1d18:1 signal intensity in molecular images, hardly any GM1d20:1 signal could be observed visually (**Figure 5B**). Both species were expressed at relatively low levels in the VPM, leading to a significantly higher GM1d18:1/GM1d20:1 ratio in the DMN (4.53 ± 0.12, *n* = 18) versus the VPM (3.14 ± 0.13, *n* = 18, Mann–Whitney test, *p* < 0.0001, **Figure 5C**). Differential expression was also observed within the lateral posterior thalamic nucleus (**Figures 5A,B**, not quantified).

### GM1d18:1/d20:1 Ratio within the Hypothalamus and Amygdala

**FIGURE 6:**
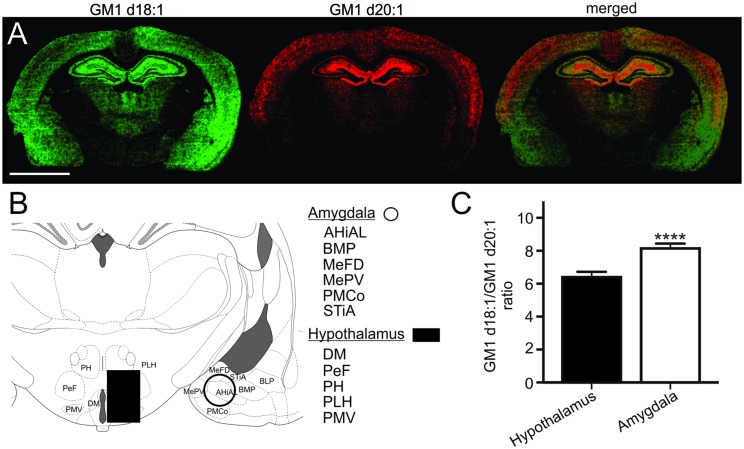
**Expression images of GM1d18:1 (green) and GM1d20:1 (red) showed relatively high GM1d18:1 expression within the amygdala, while GM1d20:1 expression was low within the hypothalamus and amygdala **(A)**.** Scale bar = 4 mm. A schematic cross section shows regions included in ROIs from which mass spectra were generated for the amygdala and hypothalamus **(B)**. AHiAL, amygdalohippocampal area, anteriolateral part; BMP, basomedial amygdaloid nucleus, posterior part; BLP, basolateral amygdaloid nucleus, posterior part; DM, dorsomedial hypothalamic nucleus; MePD, medial amygdaloid nucleus, posterodorsal part; MePV, medial amygdaloid nucleus, posteroventral part; PeF, perifornical nucleus; PH, posterior hypothalamic nucleus; PLH, peduncular part of lateral hypothalamus; PMCo, posteromedial cortical amygdaloid nucleus; PMV, premammillary nucleus, ventral part; STiA, bed nucleus of the stria terminalis, amygdaloid division. The GM1d18:1/GM1d20:1 ratio was significantly higher within the amygdala than the hypothalamus (Mann–Whitney test, ^∗∗∗∗^*p* < 0.0001, **C**).

Based on the relative expression of both GM1 species in molecular images, the hypothalamus had a relatively lower content of GM1 in comparison to the thalamus, the hippocampus, cortical regions and the amygdala (**Figure 6A**). While the GM1d20:1-species was hardly visible within the hypothalamus and amygdala, GM1d18:1 showed a markedly higher expression within the amygdala. When quantified (**Figure 6B**), GM1d18:1 expression was eight times higher than GM1d20:1 expression in the amygdala (8.15 ± 0.29, *n* = 19), the highest ratio measured in any brain region analyzed (**Figure 6C**). This ratio is significantly higher than the ratio in the hypothalamus, where there was more than 6 times more GM1d18:1 than GM1d20:1 (6.40 ± 0.33, *n* = 18, Mann–Whitney test, *p* < 0.0001, **Figure 6C**).

## Discussion

### Biological Role of the Ceramide Moiety of Gangliosides

Studies focusing on the ceramide moiety of gangliosides in the brain are relatively scarce, but important discoveries reported recently point toward a functionally distinct role for these different ganglioside species (Sugiura et al., 2008; Whitehead et al., 2011; Oikawa et al., 2015). Based on the biochemical differences between d18:1- and d20:1-sphingosine molecules in the ceramide base, it has been suggested that the different species influence local membrane fluidity differentially, with a longer sphingosine tail making the membrane flatter and more rigid (Sonnino and Chigorno, 2000). Membrane fluidity can determine the three dimensional environment of specialized membrane microdomains, such as lipid rafts, ultimately influencing the accessibility of membrane proteins and the occurrence of protein–protein interactions (Sonnino et al., 1990). How these biochemical features translate into biological function is at this point not known for ganglioside GM1. Yet, GM1d18:1 and GM1d20:1 species have been found to be upregulated after severe cerebral ischemia, each GM1 species following a slightly different expression pattern in and around the infarct (Whitehead et al., 2011). In the same study, the spatio-temporal expression profiles of d18:1 and d20:1 species of GD1, GT1, GM2, and GM3 were also specific to the sphingosine chain length following ischemia. The biological relevance of the ceramide moiety of gangliosides was recently confirmed in a study where the c18:0 and c20:0 fatty acid species of GD1b were shown to have differential effects on the assembly of amyloid protein (Oikawa et al., 2015). Likewise, GM1d18:1 and GM1d20:1 were upregulated to slightly different degrees in a combined model of Amyloid Beta load and striatal ischemia (Caughlin et al., 2015). These findings, together with the unique biochemical properties of different sphingosine carbon chain lengths and the tight regulation of their expression emphasize a potential functionally meaningful role of the ganglioside ceramide bases in health and disease of the central nervous system.

From past investigations into the expression of the c20-sphingosine species of gangliosides we know that the expression of this molecule increases during development and with age. We therefore chose a rat model that would reflect a mature brain state. Aging rats to 8–10 months, which corresponds to a young to middle-aged adult, ensures that GM1d20:1 is substantially expressed. Brains at this level of maturity likely reflect the general anatomical expression pattern of GM1d18:1 and GM1d20:1 through most of adulthood, as our findings of hippocampal expression ratios are in line with previous reports in mice (Sugiura et al., 2008). However, Sugiura and colleagues found an increase in c20-sphingosine containing GD1 in the molecular layer of the DG between 2 and 33 months old mice, suggesting a slow but potentially continuous increase of the c20-sphingosine species of gangliosides with age. Investigations at more senior stages in life are needed to further our understanding of how the GM1d18:1/GM1d20:1 ratio changes in the aging and senescent brain, and whether various anatomical regions undergo different changes at different times as the brain ages.

### Discussion of Present Findings in Anatomical Context

In the somatosensory cortical regions analyzed, the superficial layers, which are part of a mostly corticocortical projection network, have a distinctly lower GM1d18:1/GM1d20:1 ratio than deeper layers. GM1d20:1 is highly expressed in superficial and intermediate layers, which are projection targets for thalamic input (Hooks et al., 2013; Shigematsu et al., 2015). Whether thalamic axons are a main source of GM1d20:1 in these regions can at this point only be speculated. While our analysis was focused on parietal cortical areas, where gradients in GM1d18:1 and GM1d20:1 expression were obvious in the molecular images, other cortical areas seem to diverge from this pattern. More comprehensive studies on the comparison of different cortical areas may address the question whether ganglioside expression relates to the unique neuronal structures known to exist in specialized cortical areas particularly in primates (Elston and Fujita, 2014), but also in mice (Ballesteros-Yanez et al., 2006).

Measurements of GM1d18:1 and GM1d20:1 in the corpus callosum and the internal capsule demonstrate that these two major white matter regions differ in lipid expression. The corpus callosum contains mostly commissural fibers that connect one hemisphere with the other, while the long projection fibers that connect more distant brain regions with each other are concentrated within the internal capsule. The differential GM1 profile in these regions may reflect the differences in myelination, as the internal capsule is myelinated earlier than the corpus callosum and its myelin sheath is generally thinner than that of callosal axons (Paus et al., 2001).

Among all subregions of the hippocampus included in the present analysis, the granule cell layer of the DG demonstrated the highest GM1d18:1/GM1d20:1 ratio. The thin subgranular zone of the DG is one of the few locations in the mature central nervous system where adult neurogenesis occurs. Although we could not distinguish the subgranular zone in our mass spectrometry images, we cannot exclude that this zone was sampled together with the more mature GC. Having a pool of neural stem cells and immature newborn neurons in the ROI may influence the spectrum toward a higher GM1d18:1/GM1d20:1 ratio as immature neurons do not express notable amounts of GM1d20:1.

An almost complementary expression of GM1d18:1 and GM1d20:1 was seen in the molecular layer of the DG (high GM1d20:1) versus the stratum radiatum of the CA1 and CA3 regions (high GM1d18:1). This observation mirrors what has been reported for the mouse hippocampus previously (Sugiura et al., 2008). As the authors of that article suggest, the high GM1d20:1 content within the molecular layer can be explained by projection fibers from entorhinal cortex, which terminate in this region of the DG. Using MALDI IMS, the authors show that entorhinal cortical neurons indeed express significant amounts of GM1d20:1 (Sugiura et al., 2008). This is a prime example for the fact that the GM1 content in any given region is a composite of somatic, axonal, dendritic, and oligodendrocytic (myelin) expression of GM1. Therefore, the GM1d18:1/GM1d20:1 ratio has to be carefully interpreted for each anatomical region, considering the connectivity to and from the area.

When focusing on the relative expression patterns of GM1d18:1 and GM1d20:1visible in images, it is interesting to note that both GM1 species are not commonly highly expressed in the same area. Most often, either moderate expression of both species or almost complementary expression is observed. A pattern of expression emerges when comparing phylogenetically older structures, such as the piriform cortex that processes olfactory information and the hypothalamus that controls metabolism throughout the body, with phylogenetically younger structures such as the somatosensory cortex (Hofman, 2014; Luzzati, 2015). As organisms are known to replicate the phylogeny of their animal species during their own development or ontogeny (Shaw et al., 2008), it is an interesting idea that the advent of GM1d20:1 at later stages during ontogeny may reflect a more recent appearance of d20:1 ganglioside species on the evolutionary tree. While merely speculative, this hypothesis should be testable by analyzing d20:1 ganglioside content of brains from evolutionarily “older” animal species such as turtles (Northcutt, 2002).

### Considerations for Interpreting MALDI IMS Data

**FIGURE 7:**
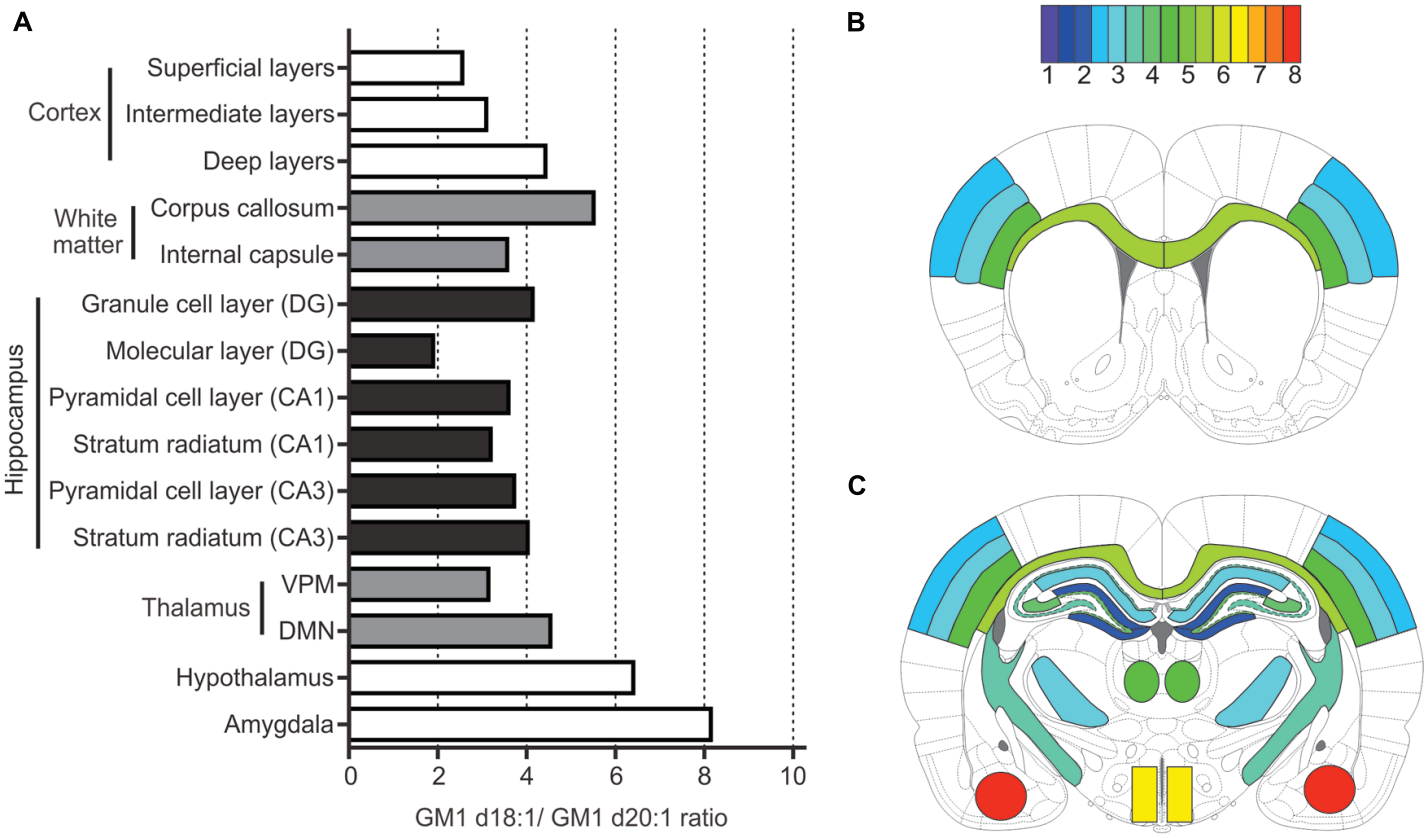
**Summary of quantification for all anatomical areas.** Bar graph provides an overview of expression means for GM1d18:1/GM1d20:1 ratios across all regions measured **(A)**. The GM1d18:1/GM1d20:1 expression ratio is color coded for all analyzed brain regions in schematic cross sections from anterior bregma **(B)** and posterior bregma **(C)**.

Using MALDI IMS, we here report a wide variety of anatomically specific expression ratios of GM1d18:1/GM1d20:1 (**Figure 7**), which likewise points toward a differential functional role of these lipid species. When interpreting mass spectrometric data from imaged regions, one should be mindful that GM1 ganglioside is preferentially incorporated into lipid rafts, and therefore the overall measured ratios in each anatomical region include synaptic GM1. Pre-synaptic GM1 may be expressed by projection neurons from remote brain areas that synapse with resident neurons, and post-synaptic GM1 may include dendrites from neighboring neurons that reach into the ROI where the GM1 ratio was measured. Therefore, the GM1d18:1/GM1d20:1 ratio measured in these areas does not necessarily reflect the expression profile of resident neurons.

Another limitation of MALDI IMS that has to be kept in mind is that molecular images acquired at this resolution do not allow discrimination of cytoarchitectonic features. Such cytoarchitectonic features clearly delineate borders between different anatomical regions, including cortical subregions (Brodmann, 1909). As a consequence of this limitation, we rely on the Rat Brain Atlas by Paxinos and Watson (1998) for identification of ROIs.

A different consideration has to be taken into account as well when using MALDI IMS. One of the limitations of this technique is that each mass spectrum obtained from individual images is unique in signal intensity and signal to noise ratio based on slight differences in tissue properties, tissue harvest and preparation, matrix application, and other inevitable variables (Heeren et al., 2009). Therefore, absolute quantification requires reference to concentration standards spotted on the tissue, which is increasingly being explored for exogenous compounds such as drugs (Nilsson et al., 2010; Lietz et al., 2013). Because we are measuring endogenous molecules, we can quantify expression of gangliosides by relating either one peak to a different peak in the same spectrum, as done in the present work, or by relating an individual peak in one anatomical area to the corresponding peak in a different anatomical area within the same image (Caughlin et al., 2015). Thus, the sample-to-sample variations in IMS signal intensity due to sample preparation and instrumentation should be canceled out by reporting ratios of GM1d18:1/GM1d20:1 expression rather than absolute measures of expression. Absolute quantification of GM1 species using MALDI IMS is additionally confounded by potential sialic acid breakdown from polysialylated gangliosides such as GD1, GT1, and GQ1, a process that increases the signal of the monosialylated GM1 (Ivleva et al., 2004).

## Conclusion And Outlook

Matrix-assisted laser desorption/ionization IMS allows us to discover new, potentially meaningful insights into the chemical make-up of the brain. The tight regulation of expression of GM1d18:1 and GM1d20:1 in anatomically distinct regions as described here points toward differential functional properties of these two species. With novel technologies and instrumentation at hand, investigations into the expression of gangliosides with difference ceramide moieties in pathological conditions will help elucidate what role these molecules play in injury, in disease and for the challenges faced by the aging brain.

Based on the distinct anatomical expression pattern reported here, questions arise as to how expression of GM1d20:1 and GM1d18:1 is so tightly regulated. Elucidation of the genetic and enzymatic control of expression may advance our understanding of these molecules greatly. Specifically, taking into account the increase of GM1d20:1 expression during development, GM1d20:1 may play a unique role in higher order brain functions. In addition, elucidating the expression pattern of GM1d20:1 in the aging brain may help understand whether this molecule may be involved in making the aging brain more vulnerable to degenerative challenges.

## Author Contributions

NW conceived the studies, carried out the experiments, analyzed the data and wrote the manuscript SC developed the methodology and analyzed the data KY developed the methodology SW conceived the studies, analyzed the data, wrote the manuscript and directed the research program.

## Conflict of Interest Statement

The authors declare that the research was conducted in the absence of any commercial or financial relationships that could be construed as a potential conflict of interest.

## ABBREVIATIONS

APP: amyloid precursor protein
AUC: area under curve
CA: cornu ammonis
DG: dentate gyrus
DMN: dorsomedial nucleus of thalamus
ET-1: Endothelin-1
GC: granular cell layer
GM: monosilylated ganglioside
GD: disialylated ganglioside
GT: trisialylated ganglioside
IMS: imaging mass spectrometry
MALDI: matrix-associated laser desoprtion/ionization
PC: pyramidal cell layer
TG: transgenic
VPM: ventroposteriormedial nucleus of thalamus
WT: wildtype
